# Stability of the effect of silencing fibronectin type III domain-protein 1 (*FN3D1*) gene on *Anopheles arabiensis* reared under different breeding site conditions

**DOI:** 10.1186/s13071-020-04078-2

**Published:** 2020-04-19

**Authors:** Serkadis Debalke, Tibebu Habtewold, George K. Christophides, Luc Duchateau

**Affiliations:** 1 Department of Medical Laboratory Science & Pathology, Jimma University, Jimma, Ethiopia; 2 Biometrics Research Group, Ghent University, Ghent, Belgium; 3 Department of Life Sciences, Imperial College London, London, UK

**Keywords:** *Anopheles arabiensis*, Larval breeding sites, Gene silencing stability, Survival, *FN3D1* gene

## Abstract

**Background:**

Malaria vector mosquitoes acquire midgut microbiota primarily from their habitat. The homeostasis of these microbial communities plays an essential role in the mosquito longevity, the most essential factor in the mosquito vectorial capacity. Our recent study revealed that silencing genes involved in regulation of the midgut homeostasis including *FN3D1*, *FN3D3* and *GPRGr9* reduced the survival of female adult *Anopheles arabiensis* mosquitoes. In the present study, we investigate the stability of the gene silencing efficiency of mosquitoes reared in three different breeding conditions representing distinct larval habitat types: town brick pits in Jimma, flood pools in the rural land of Asendabo and roadside pools in Wolkite.

**Methods:**

First-instar larvae of *An. arabiensis* mosquitoes were reared separately using water collected from the three breeding sites. The resulting adult females were micro-injected with dsRNA targeting the *FN3D1* gene (AARA003032) and their survival was monitored. Control mosquitoes were injected with dsRNA *Lacz*. In addition, the load of midgut microbiota of these mosquitoes was determined using flow cytometry.

**Results:**

Survival of naïve adult female mosquitoes differed between the three sites. Mosquitoes reared using water collected from brick pits and flood pools survived longer than mosquitoes reared using water collected from roadside. However, the *FN3D1* gene silencing effect on survival did not differ between the three sites.

**Conclusions:**

The present study revealed that the efficacy of *FN3D1* gene silencing is not affected by variation in the larval habitat. Thus, silencing this gene has potential for application throughout sub-Saharan Africa.
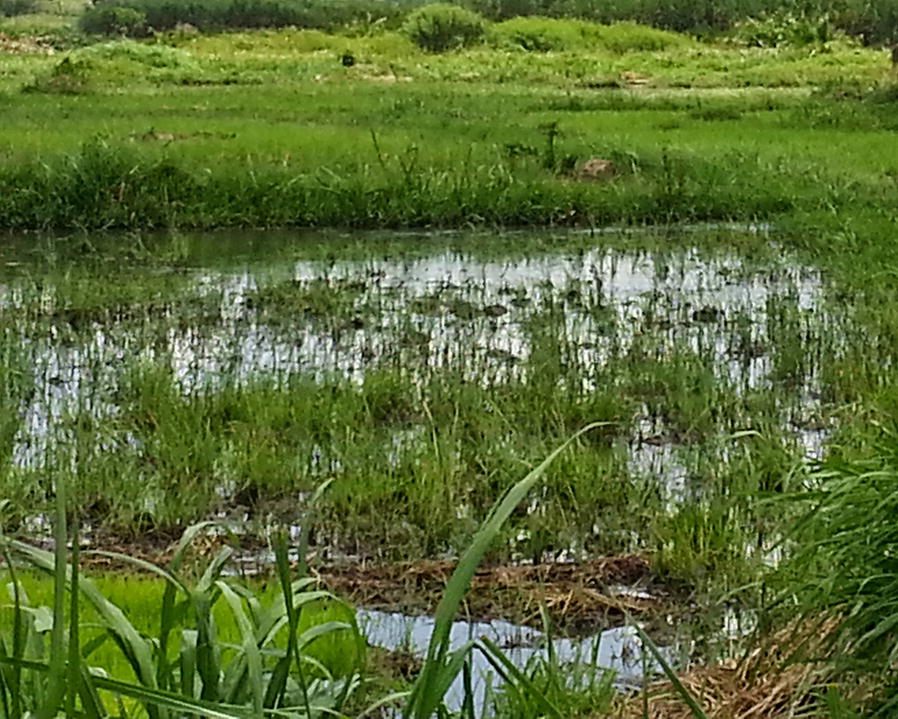

## Background

Malaria still thrives in sub-Saharan Africa. The region holds over 90% of the 219 million malaria cases with an estimated 435,000 malaria deaths in 2017 [1]. This disproportionate share is due to the principal malaria vectors in the region that exhibit a higher vectorial capacity, i.e. *Anopheles gambiae* (*s.s*), *Anopheles coluzzii*, *Anopheles arabiensis* and *Anopheles funestus*. Key parameters for vectorial capacity include preference to feed on humans, susceptibility to *Plasmodium* infection and longevity of the mosquito. It can take two weeks and beyond for the *Plasmodium* parasites to complete the mosquito stage development after which they can be transmitted to another human host. On the other hand, in tropical regions, the average lifespan of *Anopheles* mosquitoes is 14 to 19 days [2, 3], resulting in only a few long-living vectors that can transmit malaria. Therefore, a promising strategy to eliminate malaria transmission is to reduce the mosquito lifespan, as even a small reduction would have a large impact on transmission [4].

Adult mosquito survival is influenced by the environment during the preceding immature stage. Environmental factors consist of the physicochemical characteristics of larval habitats [5, 6], and the quality and availability of nutrients [7, 8]. Previous studies have demonstrated that larval fitness has a major effect on the adult survival of different malaria mosquitoes [8–11]. For instance, female adult mosquitoes maintained in a high nutritive larval environment lived longer than in a poor nutritive environment [9, 12]. A nutritionally restrictive larval environment yields mosquitoes with reduced size with significantly shorter survivorship [9, 10]. Microorganisms and organic materials in the larval pool are the major constituents of the larval diet [13]. Previous studies have demonstrated that bacteria in the breeding water are most critical for the quality of the larval diet and their absence leads to increased larval mortality [14–17].

An increasingly important factor that affects the life traits of adult mosquitoes is the pollution of the larval habitats. Rapid and unplanned expansion of urbanization in sub-Saharan Africa increases pollution of the surface waters with domestic or industrial discharges of untreated effluents [18, 19]. *Anopheles* larvae have shown a high resilience to the high toxicity of some of these wastes in the urban and suburban sites, but the impact of such pollution on the life traits of the resulting adult mosquito is poorly understood. However, there is speculation that exposure of larvae to toxins dissolved in the water might contribute to the rising prevalence of vector resistance to pyrethroids in the cities across the region [20, 21]. It was also suggested that larvae developing in polluted water can lead to a significant fitness cost [22].

Based on the above facts, we assessed the survival of adult female *An. arabiensis* mosquitoes reared in water collected from three different breeding sites. In our previous study we observed that silencing the midgut gene fibronectine type III domain-protein 1 (*FN3D1*) involved in the midgut homeostasis reduced the longevity of *An. arabiensis* mosquitoes [23]. Therefore, in the present study we further assessed the stability of the *FN3D1* gene silencing effect on *An. arabiensis* mosquitoes reared in water collected from three different breeding sites.

## Methods

### Study sites and sample collection

**Fig. 1:**
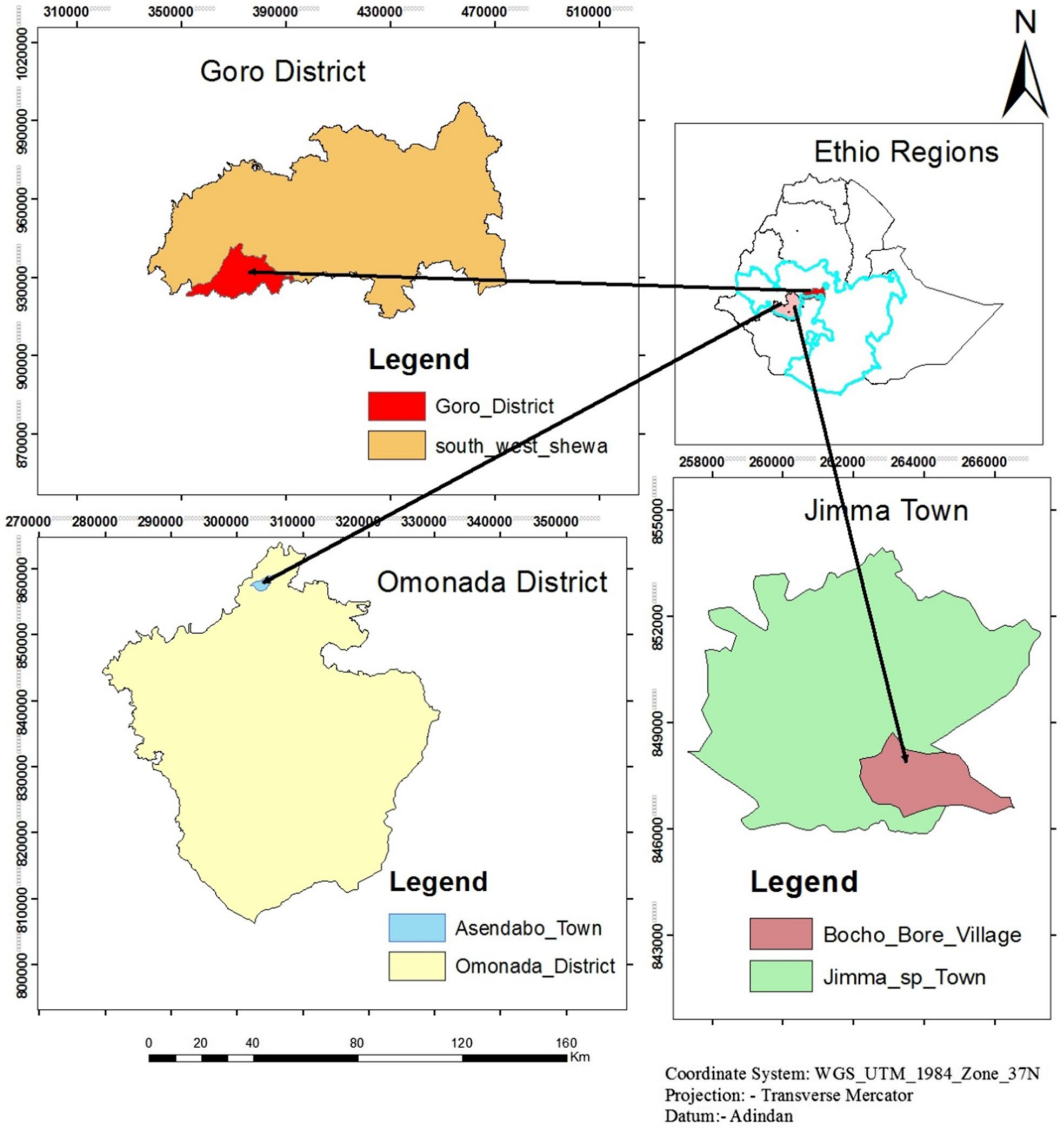
Map showing larval breeding sites where rearing water samples were collected

Water samples for mosquito rearing were collected from three malaria endemic sites including Wolkite, Jimma and Asendabo, south-west Ethiopia (70°40′0.01″N, 36°49′59.99″E) (Fig. 1). From each site, a single pool confirmed to support *An. arabiensis* larvae was selected to collect rearing water using a clean jug and plastic container/jerry cans. Simultaneously water samples for bacterial counts were collected using sterile, screw cup bottles following standard operational procedures [24]. The water collected for rearing purposes was filtered using a fine mesh linen cloth in order to remove debris and mosquito eggs and larvae. The filtered water was then transferred directly to clean larvae rearing pan or boiled for 10 min and cooled before transferring to the rearing pans.

### Analysis of water from selected sites

pH, conductivity and dissolved oxygen was measured on site using a Portable Multi meter (HQ40D, HACH). Salinity was measured using a salinity meter (TRACER PocketTester) and turbidity with a turbidity meter (Wag-WT 3020, Wagtech International, Gateshead, UK). Total dissolved solids (TDS) and total suspended solids (TSS) were measured by the gravimetric method. In brief a volume of 100 ml of well-mixed water samples was filtered using a glass-fiber-filter with applied vacuum. The filtered samples were then washed with three successive 10 ml volumes of distilled water which permitted complete drainage between washings. The suction was continued for about 3 min after filtration was completed. The filtrate (with washing) was then transferred to a weighed evaporating dish and evaporated to dryness on a steam bath. If the filtrate volume exceeded dish capacity, successive portions were added to the same dish after evaporation. Finally, the samples were dried for at least 1 h in an oven at 180 ± 2 °C for TDS and 103–105 °C for TSS, allowed to cool in a desiccator to balance temperature, and weighed [24]. All the parameters were measured based on the supplier’s guidelines and the instruments were calibrated before the samples analysed. A total of two replicates was taken for each site.

The total bacterial count was assessed for the water samples using membrane filter techniques. For each sample tenfold serial dilution was prepared with a total volume of 100 ml and allowed to pass through a funnel covered with a membrane filter (with a size of 47mm diameter by 0.45 µm porosity) in order to trap the bacteria available in the water. Then the membrane with the trapped bacteria was placed in a Petri dish containing a pad saturated with sterilized M-lauryl sulfate broth (Sigma-Aldrich, Bangalore, India). The M-lauryl sulfate broth media was prepared according to the manufacturer’s guidelines. The passage of nutrients through the filter during incubation facilitates the growth of organisms in the form of colonies on the upper surface of the membrane. Then, the inoculates were incubated at a temperature of 37 °C for 24 h where after the total number of the colonies was counted using a magnifying lens [24].

### Mosquito rearing

*Anopheles arabiensis* obtained from a laboratory strain of the Adama Malaria Research Centre, Ethiopia, was used in this study. Recently hatched first-instar larvae were reared in the water collected from the three breeding sites. Two-hundred larvae from the same batch of eggs were dispensed into the rearing trays and supplied with an equal amount of a mixture of yeast and fish food. The rearing water was changed every two days and development of larvae was monitored daily. Next, the enclosed pupae were counted and transferred into 10 ml glass beakers independently and kept in adult cages (W15 × D15 × H15 cm, BugDourm, Watkins & Doncaster, Leominster, UK) until the emergence of the last pupae. The emerged adults were maintained on 10% sugar at 27 °C, 70% humidity with 12:12 h light:dark photocycle [25]. The experiment was done in triplicate.

Adult wing size was measured to evaluate differences in the adult female body size. For this experiment, 10 individual mosquitoes were removed from each adult cage to measure their wing size as follows: a single wing was clipped from each mosquito using scalpel blade and mounted onto a glass microscope slide. Then the wing size was measured using a micrometer eyepiece with stereomicroscope. Measurements were taken from the tip of the wing (excluding fringe) to the distal end of the alula [26].

### Gene silencing and survival assay

From each breeding site, 15–24 adult female mosquitoes were CO_2_ anesthetized and treated with 69 µl of the *FN3D1* dsRNA gene and another group with non-mosquito ds*LacZ* gene. Double stranded RNA (DsRNA) for *FN3D1* and the control *LacZ* gene was prepared from complementary DNA (cDNA) as described in our previous study [23]. The cDNA was synthesized from 1 μg of the tRNA using Prime-Script™ 1st-strand cDNA Synthesis Kit (TaKaRa, Saint-Germain-en-Laye, France) which was extracted from 10 whole female *An. arabiensis* mosquitoes using TRIzol reagent (Invitrogen, Carlsbad, USA). The following primer sequences tailed with the T7 promoter were used to synthesize the two dsRNA: *FN3D1* (forward: GAT GGA CGT GGA TCA GCC; reverse: TGG ATC GTC CTC ATC ACT GT) and *LacZ* (forward: AGA ATC CGA CGG GTT GTT ACT; reverse: CAC CAC GCT CAT CGA TAA TTT).

On day four, after the mosquitoes were starved overnight, they were fed on blood. At 24 h post-blood-meal, 5 mosquitoes were sampled for each breeding site independently for both unboiled and boiled water and their midguts were dissected. The midgut samples were then homogenized in 100 µl 4% paraformaldehyde (PFA) in phosphate-buffered saline (PBS). The number of bacteria was then counted using flow cytometry from five pooled midgut samples per replicate [27]. A total of 3 replicates of microbiota analysis were performed.

The survival of mosquitoes was monitored starting from 24 h post-injection for 20 days. All mosquitoes were supplied with 10% sugar solution and monitored daily, and they obtained blood meal at 4-day intervals post-injection. Three replicates each consisting of 15–24 mosquitoes were performed.

### Data analysis

Data analysis was performed using the statistical software package R version 3.3.2. Survival of the *FN3D1* gene- and *LacZ* gene-silenced mosquitoes and the naïve mosquitoes with boiled and unboiled water were depicted by Kaplan Meier survival curves as a function of site. The effect of gene silencing on survival was modelled by the Cox proportional hazards frailty model [28], with replicate as frailty term and including as covariates the gene (the *FN3D1* target and *LacZ* control gene), the site (Jimma, Asendabo and Wolkite) and the two-way interaction. The effect of site and boiling in the naïve mosquitoes was analysed in the same way. The hazard ratio was used as a summary statistic, together with the median time to death. The bacterial counts were first log-transformed and then compared by a mixed model with replicate as random effect and the *F*-test was used to compare the silencing of the *FN3D1* target gene and the control *LacZ* gene on the one hand, and the boiling on the other hand. The ratio of the bacterial counts in the control *LacZ* gene and the *FN3D1* target gene was used as a summary statistic. The t-test was used to compare the number of emerged adults and pupae and the adult wing length between the three breeding sites. All tests were performed at a significance level of 5%.

## Results

### Analysis of water samples

**Fig. 2:**
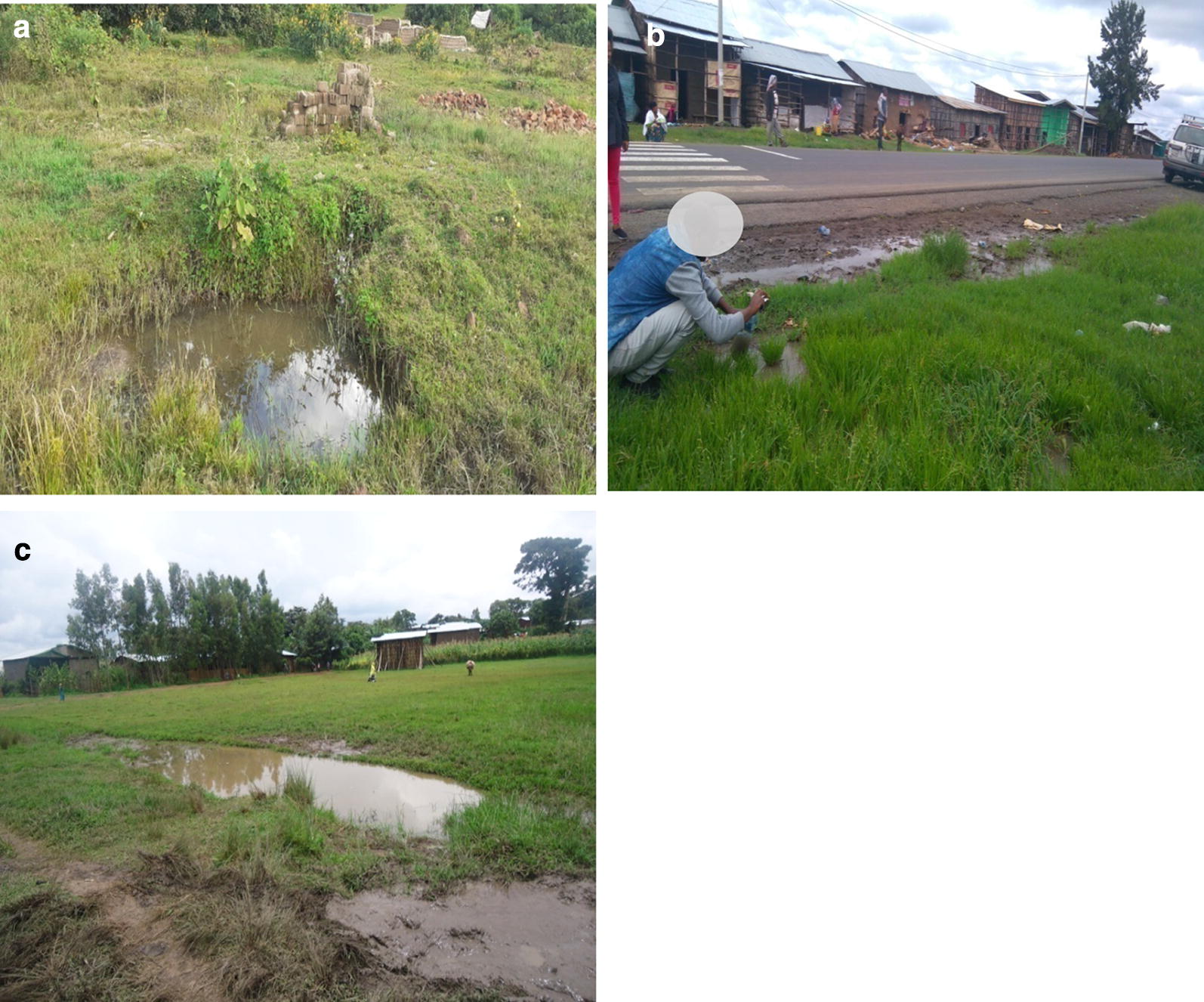
Breeding sites from where larval rearing water samples collected representing brick-pit pool at Jimma town (**a**), flooded farmland at Asendabo (**b**) and roadside pool at Wolkite (**c**)

Pictures of the three breeding sites are shown in Fig. 2 and their physicochemical characteristics are provided in Table 1. The water samples from Jimma and Asendabo had close to the neutral pH, while water samples from Wolkite were alkaline with the highest salinity. Nevertheless, the pH for all the sites was within a range that was previously reported suitable for the natural breeding habitats of *An. arabiensis* [29]. The level of dissolved oxygen was considerably higher for Wolkite and Asendabo compared to Jimma. The Asendabo had also the highest total bacterial count compared to Jimma or Wolkite.

**Table 1:** Physicochemical characteristics and bacterial count of water samples from the three sites

Parameter	Wolkite	Jimma	Asendabo
pH	8.2 (7.9–8.5)	7.3 (7.1–7.5)	7.15 (7.0–7.4)
Salinity (ppm)	225 (180–320)	85 (75–125)	160 (100–175)
DO (mg/l)	7.2 (2.3–7.6)	4.5 (1.8–8.8)	6.3 (5.1–9.0)
Turbidity	27.3 (4.9–80.2)	51.7 (2.2–87.1)	64.3 (47.2–146.0)
TDS (mg/l)	507.5 (256.0–665.5)	237.0 (185.5–317.0)	167.0 (163.0–256.2)
TSS (mg/l)	157.5 (72.0–358.0)	275.5 (156.0–293.0)	170.0 (151.0–323.0)
Chlorophyll a	11.6 (11.3–12.0)	11.9 (11.7–12.3)	12.2 (12.0–12.4)
Bacterial count (10^3^ CFU/100 ml)	317 (233–400)	233 (192–291)	546 (252–660)

*Note*: The data are presented as median (range)

*Abbreviations*: DO, dissolved oxygen; TDS, total dissolved solids; TSS, total suspended solids

### Larval development

Our results demonstrated that, generally, the larvae reared with water from Asendabo performed better when compared to the rest of the sites (Table 2). For instance, the pupation rate for Asendabo (85.8%) was significantly higher compared to Wolkite (68.5%) (*t* = 5.69, *df* = 4, *P* = 0.005) but not to Jimma (78%) (*t* = 5.69, *df* = 4, *P* = 0.062). No significant differences of percentage of pupae developed in adult mosquitoes could be found between Asendabo, Wolkite and Jimma. The mean adult wing length was significantly larger for Asendabo (3.2 mm) compared to Jimma (3.0 mm) (*t* = 2.58, *df* = 87, *P* = 0.012) and Wolkite (2.7 mm) (*t* = 8.23, *df* = 87, *P* < 0.001). Together, these results suggest that the above parameters may be positively impacted by the level of dissolved oxygen and bacterial count in the breeding water. Accordingly, the water from Asendabo is considered most suitable for larval development.

**Table 2:** The rates of pupation and adult emergence (*n* = 3 batches of 200 eggs), and the mean adult wing size (*n* = 30) in mosquitoes reared in the water collected from the three sites

Breeding site	Pupa (%)	Adult (%)	Mean wing size (mm)
Wolkite	68.8	93.7	2.7
Jimma	78.2	93.6	3.0
Asendabo	85.8	95.9	3.2

### Survival rate of adult mosquitoes

**Fig. 3:**
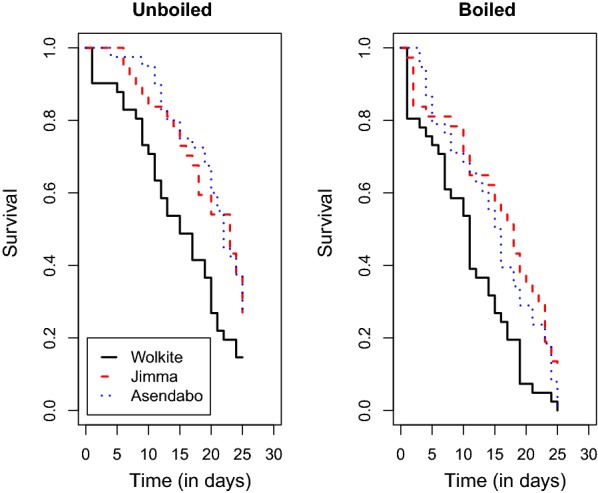
Survival as a function of time for the naive mosquitoes for the three sites (Wolkite, Jimma and Asendabo) and water boiled or unboiled

**Fig. 4:**
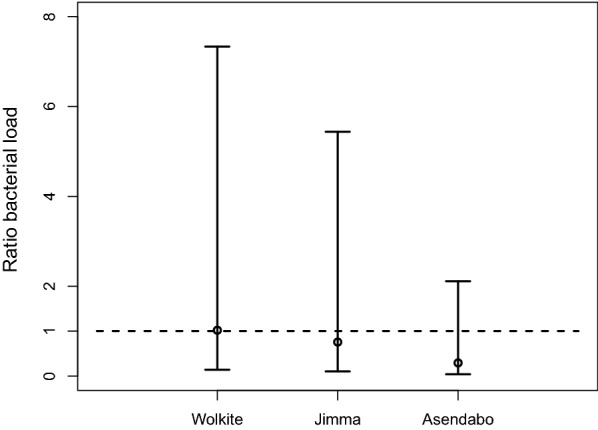
Ratio of adult gut bacterial load (95% confidence interval) of mosquitoes in boiled *versus* unboiled water in the three sites

Survival of the mosquitoes is depicted as a function of time in Fig. 3 for the different settings, i.e. different sites and water boiled or not. There was no significant interaction between the site and boiling effect, i.e. the effect of boiling the water has a similar effect for the three sites. A significant effect of boiling the water (*χ*^2^ = 17.2, *df* = 1, *P* < 0.001) and an almost significant difference between the sites (*χ*^2^ = 5.64, *df* = 2, *P* = 0.059) was found. The hazard ratio of the mosquitoes grown in boiled water compared to unboiled water was 2.25 (95% CI: 1.42–3.56). The hazard ratio of Jimma compared to Wolkite was 0.53 (95% CI: 0.32–0.88) whereas the hazard ratio of Asendabo compared to Wolkite was 0.51 (95% CI: 0.31–0.84). The adult gut bacterial count ratios in mosquitoes grown in the unboiled *versus* the boiled water for the three sites are presented in Fig. 4.

### Effect of the *FN3D1* gene silencing

**Fig. 5:**
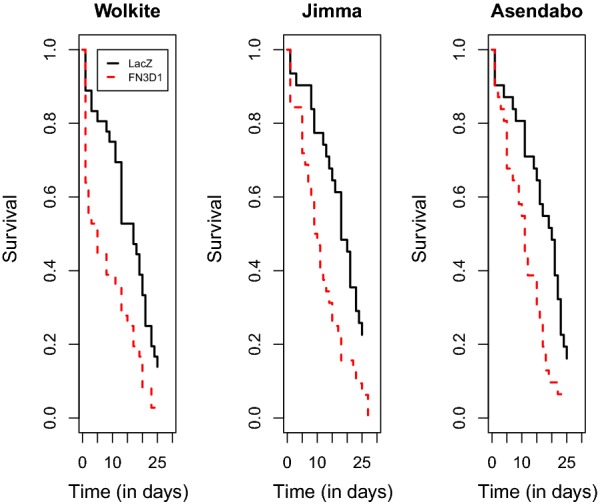
Survival as a function of time for the control *LacZ* and target *FN3D1* gene silenced mosquitoes at the three sites

Survival of the *FN3D1-* and *LacZ-*silenced mosquitoes is depicted as a function of time in Fig. 5 for the different sites. There was no significant interaction between the gene silencing effect and the site, i.e. the effect of silencing was the same for the three sites. Both gene silencing (*χ*^2^ = 25.7, *df* = 1, *P* < 0.001) and site (*χ*^2^ = 8.15, *df* = 2, *P* = 0.017) had a significant effect on survival. The hazard ratio of the *FN3D1-*silenced mosquitoes compared to the *LacZ-*silenced mosquitoes was 1.96 (95% CI: 1.58–2.43). The hazard ratio of Jimma compared to Wolkite was 0.69 (95% CI: 0.54–0.89) whereas the hazard ratio of Asendabo compared to Wolkite was 0.86 (95% CI: 0.67–1.10).

**Fig. 6:**
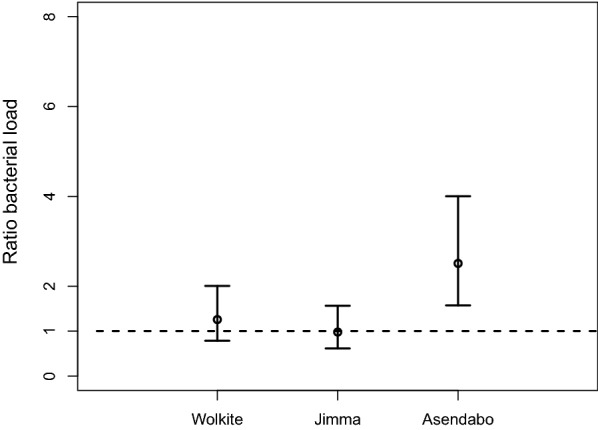
Ratio of adult gut bacterial load (95% confidence interval) in *FN3D1-*silenced *versus LacZ-*silenced mosquitoes in the three sites

Significant effects for the microbiota were found for the gene silencing (*F*_(1, 14)_ = 7.59, *P* = 0.016) and for the interaction between gene silencing and site (*F*_(2, 14)_ = 4.23, *P* = 0.038). Therefore, we study the gene effect at each location separately. A significantly higher bacterial load in the *FN3D1-*silenced mosquitoes as compared to the *LacZ-*silenced mosquitoes was only found for Asendabo, with a ratio equal to 2.50 which differs significantly from 1 (*F*_(1, 14)_ = 14.21, *P* = 0.002) (Fig. 6).

## Discussion

In the present study, we demonstrated that the larval breeding habitats in the three sites vary distinctly with regard to their physico-chemical characteristics and microbial abundance. Previous studies have demonstrated that the physico-chemical characteristics [30–32] of the larval breeding habitat determine the larval and pupal density, the size and number of emerged adults and the survival of both larvae and adult mosquitoes. Thus, the mosquitoes in the study sites could also differ in their fitness (e.g. size, longevity, fecundity) and capacity to support and transmit the malaria parasite [8, 10]. Previous studies have shown that different species of mosquitoes maintained and reared in low food environments had a reduced longevity, smaller body size and lower vectorial capacity [8, 9, 31, 33, 34]. Most mosquito life traits (fitness) are affected by environmental factors, and specifically the breeding habitat [8–10, 35, 36].

The present observation of an increased total number of emerged pupae for the larvae that were reared in water collected from Asendabo is an indicator for a higher suitability of this larval habitat. For this site, two parameters that stand out include a high bacterial abundance and increased oxygenation. Previous studies have established that bacteria in the breeding habitat constitute the main food source for larvae enhancing larval growth and the productivity and survival of the adult mosquitoes [8, 9, 17]. Studies have also demonstrated that higher dissolved oxygen favours the development of *Anopheles* mosquitoes [37, 38].

In this study, wing size of the adult female mosquitoes was measured because the wing size is also an indicator for the suitability of the larval breeding environment. Our data revealed there is a marked variation in the wing size between the mosquitoes originating from different breeding sites with the Asendabo site yielding mosquitoes with the largest wing compared to Jimma and Wolkite. A similar variation in the wing size among the mosquitoes grown in different larval habitats was reported earlier for *Anopheles stephensi* [9] and *Anopheles darlingi* [8, 39]. It was concluded that such variation in the wing size is closely linked to the nutrient availability in the habitat [8, 9].

Wing size is directly correlated with survival of adult female mosquitoes, i.e. the mosquitoes with longer wing showed a higher longevity than short winged mosquitoes [9, 12, 40, 41]. Comparison of the survival of the mosquitoes reared with water from the three sites in the present work was supported by the above observation. For instance, the mosquitoes reared in the water from the Asendabo site that had the longest wing displayed the longest survival.

Previously we have demonstrated that the depletion of some midgut gene proteins including the *FN3D1*, *FN3D3* and *GPRGr9* genes markedly shorten the longevity of female *An. arabiensis* [23]. In the present study, we assessed whether the variation between breeding habitats may affect the gene silencing effect. Our survival data revealed that the effect of gene silencing was not affected by variation in the larval breeding site, i.e. for all the study sites the *FN3D1-*treated mosquitoes had a similarly reduced survival rate compared to the control *LacZ* group. Thus, the variation in breeding sites does not affect the gene silencing effect on reducing the longevity of *An. arabiensis* mosquitoes. Gene silencing induces mosquito mortality by disrupting the midgut homeostasis [23], which is evidenced also in the present study where a higher bacterial load was observed in the *FN3D1-*silenced mosquitoes as compared to the *LacZ* silenced mosquitoes.

## Conclusions

The longevity of the *An. arabiensis* mosquitoes varied between the three larval breeding sites depending on biotic as well as abiotic characteristics of the larval breeding water. However, there was no evidence that these differences compromise the gene silencing effect of the *FN3D1* gene on the mosquito survival. Therefore, interventions based on the silencing of such genes, and thus the reduction of mosquito longevity, offer a universal strategy to block malaria transmission.

## Data Availability

Datasets are available from the corresponding author upon reasonable request.

## Abbreviations

cDNA: complementary DNA
DNA: deoxyribonucleic acid
DO: dissolved oxygen
dsRNA: double-stranded RNA
*FN3D1*: fibronectin type III domain-protein 1
*FN3D3*: fibronectin type III domain-protein 3
*GPRGR9*: G protein-coupled receptor protein 9
PBS: phosphate-buffered saline
PFA: paraformaldehyde
RNA: ribonucleic acid
TDS: total dissolved solids
TSS: total suspended solids
